# Mechanistic role of APOE lipidation in Alzheimer's disease pathogenesis

**DOI:** 10.7150/thno.131926

**Published:** 2026-04-08

**Authors:** Dong Yan Zhang, Jian Wang, Nikolay V. Dokholyan

**Affiliations:** 1 Department of Neurology, University of Virginia, School of Medicine, Charlottesville, VA, USA.; 2 Departments of Neuroscience, Biomedical Engineering, Pharmacology, Microbiology, Immunology, & Cancer Biology, University of Virginia, School of Medicine, Charlottesville, Virginia, USA.

**Keywords:** APOE, lipidation, Aβ pathology, Tau pathology, Alzheimer's disease

## Abstract

The apolipoprotein E (APOE) *ε*4 allele is the primary genetic driver of late-onset Alzheimer's disease (AD), a complex neurodegenerative disorder characterized by the interplay of amyloid-β (Aβ) accumulation, tau pathology, neuroinflammation, and lipid metabolism dysfunction. Emerging evidence suggests that these pathological hallmarks are fundamentally linked to deficits in neuroplasticity and the continuous turnover of synapses. A growing body of evidence highlights APOE lipidation, a process by which APOE is loaded with lipids via cellular transporters such as ABCA1, as a key determinant of APOE function and toxicity. While lipidated APOE2 and APOE3 facilitate cholesterol transport and Aβ clearance, lipid-poor APOE4 is associated with impaired receptor-mediated clearance of Aβ, disrupted microglial function, increased neuroinflammation, and synaptic deficits. Furthermore, APOE lipidation status differentially influences tau pathology, potentially linking cholesterol dysregulation to tau hyperphosphorylation and aggregation. Here, we systematically examine the mechanistic role of APOE lipidation in AD pathogenesis, focusing on its effects on Aβ and tau pathology. We also discuss how dysregulation of APOE lipidation may serve as a central molecular mechanism connecting APOE4 to multiple pathological hallmarks of AD. This review examines how APOE lipidation is involved in amyloid-related and tau pathology in AD.

## Introduction

Alzheimer's disease (AD) pathogenesis involves multiple interconnected pathological processes, including amyloid-β (Aβ) deposition, tau pathology, neuroinflammation, and dysregulated lipid metabolism 1,2. The interaction between these processes and genetic susceptibility has attracted significant attention, as certain genetic variants may directly or indirectly exacerbate neurodegenerative processes. Among various genetic risk factors, the apolipoprotein E (APOE) genotype is recognized as the most critical genetic risk factor for sporadic AD 2. The three major APOE isoforms (APOE2, APOE3, and APOE4) show significant differences in disease risk: APOE4 carriers exhibit a significantly increased risk of disease, while APOE2 appears to have a protective effect. However, the exact molecular mechanisms through which these isoforms affect AD progression are not fully understood. A key challenge in the field is therefore to understand how APOE genotype mechanistically intersects with core pathological processes of AD, particularly those involving Aβ metabolism and synaptic dysfunction.

Aβ exists in a dynamic equilibrium of multiple aggregation states, where soluble oligomers, rather than amyloid plaques alone, are increasingly recognized as the primary drivers of synaptic dysfunction and cognitive decline 3-7. Early pathological changes of AD are not spatially uniform; they often emerge in brain regions characterized by high neuroplasticity and constant synaptic turnover, such as the locus coeruleus (LC), raphe nuclei, and the nucleus basalis of Meynert 8-11. These regions exhibit high levels of neuroplasticity and constant synaptic turnover, requiring efficient cholesterol delivery mediated by APOE 12. Impairments in lipid transport, particularly those associated with the APOE4 isoform, severely compromise this balance. Crucially, such disturbances in lipid homeostasis can dictate amyloid precursor protein (APP) processing, which shifts the balance toward amyloidogenic pathways 13-15, impair Aβ clearance mechanisms 16-19, and actively facilitate the nucleation and aggregation of toxic Aβ oligomers 20-22. Consequently, APOE-dependent lipid metabolism acts as a critical, pleiotropic upstream regulator bridging normal synaptic physiology with the full spectrum of Aβ pathology, highlighting the importance of lipidation-dependent mechanisms in modulating AD risk.

Beyond its effects on amyloid pathology, the lipidation state of APOE is increasingly recognized as a crucial modulator of tau pathology, often acting independently of Aβ. For instance, poorly lipidated APOE4 has been shown to disrupt intracellular cholesterol metabolism in glia, leading to anomalous lipid droplet accumulation that directly exacerbates neurodegeneration and accelerates the seeding and spreading of pathological tau 23,24. Furthermore, impaired lipid transport and altered APP processing can influence intracellular kinase activity, such as by increasing the generation of the APP intracellular domain (AICD), which in turn promotes tau hyperphosphorylation via kinases like GSK3β 25,26. Thus, APOE-mediated lipid dysregulation serves as a central hub that links early synaptic vulnerability not only to amyloidogenic pathways but also to the progressive accumulation and spread of tau tangles.

Here, we provide a comprehensive examination of the pivotal role of APOE lipidation in regulating both Aβ and tau pathology in AD. We first discuss the structural features of APOE isoforms and their associated genetic risks, followed by the foundational role of lipid metabolism and membrane organization in synaptic homeostasis. Next, we explore the mechanisms and functional consequences of APOE lipidation. Specifically, we detail how lipidation status not only governs Aβ production, clearance, and aggregation, but also acts independently to shape tau vulnerability by modulating isoform-specific kinase pathways, glial responses, and the pathological spreading of tau. From a translational medicine perspective, we highlight therapeutic approaches aimed at modifying APOE lipidation, such as targeting ABCA1 and LXR/RXR pathways or directly enhancing APOE lipidation. By systematically reviewing these lipidation-dependent mechanisms across the full spectrum of AD pathology, we identify promising targets for precision therapies.

## 1. Structure and Genetic Risk of APOE Isoforms

**Figure 1:**
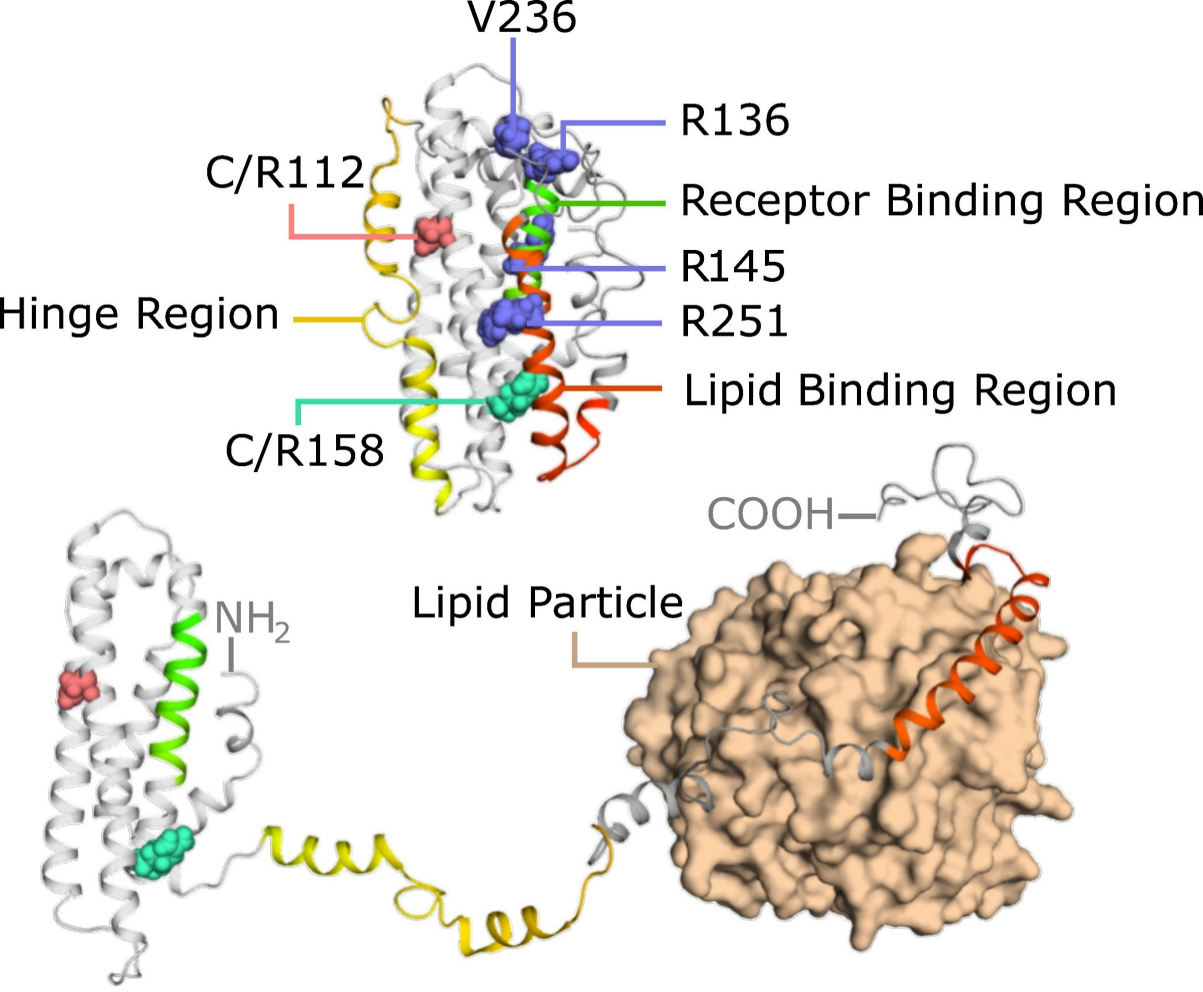
** Structural model of APOE in the lipid-free state and lipid-bound state.** APOE consists of an N-terminal helical bundle responsible for receptor binding (green) and a C-terminal domain that mediates lipid association (red). Key isoform-specific residues, C112 and R158, are highlighted (pink and cyan spheres), which influence lipid-binding affinity and receptor interaction. Upon lipid binding, the C-terminal helices reorient and anchor to the lipid surface (bottom right), facilitating a "hinge-opening" conformational change that exposes the receptor-binding region. This transition enhances APOE's ability to engage with LDL receptor family members. The structural flexibility of APOE allows it to adapt to different lipid environments, contributing to its functional diversity in lipid metabolism and neurobiology. Several rare variants are indicated: the protective APOE3-Christchurch (R136S) and APOE3-Jacksonville (V236E); the risk-reducing APOE4 (R251G); and the risk-enhancing APOE (R145C).

Understanding the structural and genetic characteristics of APOE isoforms is essential for clarifying their differential roles in AD pathogenesis 2. In humans, APOE is present in three major isoforms (APOE2, APOE3, and APOE4) that differ by only two amino acid residues at positions 112 and 158 27,28 (Figure 1). These minor structural variations, such as the substitution of arginine for cysteine, can profoundly impact the conformational dynamics and functional properties of APOE. The population distribution of these alleles exhibits striking ethnic and geographic variation 29, with APOE3 being the most prevalent (∼79% in the U.S. population), followed by the AD-risk-associated APOE4 (14%) and the protective APOE2 (7%).

From an evolutionary perspective, the APOE ε4 allele is considered the ancestral variant in humans, shared with other primates such as chimpanzees 30. The ε3 and ε2 alleles are derived variants that emerged more recently. The ε3 allele emerged about 200,000 years ago, followed by the ε2 variant between 40,000 and 10,000 years ago 31. The selection toward the ε3 allele over the ancestral ε4 state is thought to have been driven by a complex interplay of dietary shifts, climatic adaptations, and changes in pathogen exposure 30. While the "meat-eating hypothesis" suggests that ε3 provided advantages in managing the increased cholesterol intake and exposure to pathogens from carcasses associated with early human hunting 32, the migration toward different latitudes also served as a critical selective pressure 33. In extreme latitudinal environments (both high-latitude cold and low-latitude hot regions), the ε4 allele likely conferred a survival advantage by maintaining higher cholesterol levels to meet the increased metabolic demands of thermoregulation, whereas as populations settled in more temperate mid-latitudes with lower metabolic stress, the shift away from the ε4 allele likely provided a better physiological balance, ultimately reducing cardiovascular risks in modern environments 33. Furthermore, as human transitions to more stable environments reduced the burden of infectious diseases, the robust pro-inflammatory response of ε4, once protective against infant mortality and parasitic infections, became maladaptive in the context of increased longevity, contributing to chronic inflammation and late-life neurodegeneration 34. The dominance of the ε3 allele thus reflects a selection for post-reproductive health and cognitive stability across varied geographic and climatic conditions 34. Additionally, the emergence of the ε2 allele is often linked to its protective role against cardiovascular disease and its association with increased longevity, despite its complex relationship with triglyceride metabolism 35.

The structural basis for these isoform-specific effects becomes evident when examining the structural dynamics of APOE. Nuclear magnetic resonance studies reveal that APOE has a two-domain structure featuring structured helices interspersed with flexible regions, forming a versatile lipid-binding pocket between the N- and C-terminal domains (Figure 1). This structural plasticity enables APOE to undergo conformational changes upon lipid binding, exposing critical receptor interaction sites. The C-terminal domain serves as a lipid anchor, while the N-terminal helical bundle is responsible for recognizing receptors (Figure 1). Importantly, the amino acid variations distinguishing the isoforms alter the lipid-binding affinity, lipoprotein particle curvature preference, and receptor engagement efficiency. These structural and functional differences between APOE isoforms provide the molecular foundation for understanding their varying roles in lipid metabolism and AD pathogenesis.

The distinct structural features of APOE4 contribute to its pathogenic role in several neurological disorders. APOE4 has been linked to an increased risk of not only AD 2, but also Parkinson's disease 36, poor outcomes after traumatic head injury 37 and stroke 38, accelerated progression of multiple sclerosis 39, and even subtle cognitive deficits in otherwise healthy individuals 40. In the context of AD, APOE4 promotes the formation of amyloid plaques 41 and neurofibrillary tangles 42 by enhancing Aβ production and deposition, impairing Aβ clearance, reducing cholesterol delivery to neuronal membranes, and increasing tau phosphorylation and aggregation. In neuronal cultures, APOE4 inhibits neurite outgrowth, disrupts cytoskeletal and mitochondrial functions, impairs signaling pathways, and enhances Aβ-induced lysosomal leakage and apoptosis. Compared to APOE3, APOE4 fails to protect synapses from degeneration due to aging, excitotoxicity, or Aβ exposure in transgenic mouse models 43. Additionally, APOE4 is more prone to C-terminal proteolytic cleavage, depletes cytosolic androgen receptor levels, and is associated with cognitive impairments 29.

## 2. Rare Variant of APOE

The APOE-Christchurch (APOE3ch) mutation is a rare variant of the APOE3 gene, characterized by replacing arginine with serine at amino acid position 136 (corresponding to codon 154) (R136S) (Figure 1). This mutation provides a notable protection against autosomal dominant AD, and clinical studies have confirmed that it can significantly delay the onset of AD. The first case report by Arboleda-Velasquez. *et al*. showed that an individual with a pathogenic presenilin 1 (PSEN1) mutation, who also carried the APOE3ch mutation in a homozygous state, only developed mild cognitive impairment (MCI) in their 70s, delaying the typical age of onset for PSEN1 mutation carriers in that family by 30 years 44. Subsequent large-scale cohort studies further confirmed this protective effect. Among carriers of the PSEN1 E280A mutation, the median age of MCI onset in APOE3ch carriers was 52 years, compared to only 47 years in carriers without this variant, clearly demonstrating that APOE3ch can significantly delay disease onset 45. Pathologically, carriers of the APOE3ch mutation show a distinctive phenotype characterized by high amyloid burden and low tau pathology 44. The protective effect of APOE3ch involves multiple molecular regulatory mechanisms. It may act by changing the affinity of APOE for heparan sulfate proteoglycans (HSPGs) and other APOE receptors 44. In the AD brain, specific HSPGs such as agrin 46 and syndecans 47 accumulate in plaques and tangles, where they stabilize Aβ fibrils and protect them from proteolytic degradation. The APOE3ch mutant exhibits a significantly increased binding affinity to tau protein, which reduces the uptake of tau by neurons and microglia, thereby lessening the fragmentation of tau by asparagine endopeptidase (AEP). It also inhibits the release of pro-inflammatory cytokines and neurotoxicity caused by tau pre-formed fibrils or Aβ 48. APOE3ch directly protects against tau-induced synaptic loss, myelin damage, and decreases in hippocampal theta and gamma wave power by inhibiting the cGAS-STING-IFN pathway 49. Additionally, the expression of APOE3ch results in peripheral dyslipidemia, a phenotype linked to its core function of regulating microglial responses and inhibiting Aβ-induced tau seeding and spreading 50.

The APOE p.V236E variant is a rare functional variant derived from the *ε3* haplotype, and the first protective variant associated with late-onset AD to be discovered in the C-terminal lipid-binding domain of the APOE protein (Figure 1). Because its protective effect was first reported by a team in Jacksonville, Florida, USA, this variant has been named APOE3-Jacksonville (APOE3-Jac) 51. The molecular basis of this mutation is the substitution of valine with glutamic acid at amino acid position 236 of the APOE protein. This site is located within the critical protein oligomerization region at positions 230-243, which provides a structural basis for its protective effect through the regulation of protein function 52. Studies have confirmed that APOE3-Jac has significant neuroprotective value, is closely related to healthy brain aging, and can significantly reduce the risk of developing AD and Lewy body dementia 51. Meanwhile, APOE3-Jac significantly delays the onset of AD, with carriers of this variant experiencing an average delay of 10.5 years in AD onset compared to non-carriers 53. APOE3-Jac did not disrupt the normal physiological function of APOE3 53. APOE3-Jac primarily exerts its protective effects by regulating protein structure and lipid metabolism and inhibiting pathological aggregation. Specifically, the V236E mutation in the C-terminal lipid-binding domain of the APOE protein, reduces its own aggregation and enhances APOE's affinity for cholesterol and phospholipids such as phosphatidylserine and phosphatidylethanolamine 51. When expressed in astrocytes, APOE3-Jac significantly increases the levels of lipids crucial for synaptic function in the brain, promoting the formation of HDL like lipoprotein particles that bind soluble Aβ and prevent its oligomerization, while also stabilizing neuronal membranes to reduce GM1-mediated Aβ aggregation 51. Furthermore, APOE3-Jac not only reduces the levels of insoluble Aβ in the brain but also decreases the burden of Aβ fibrillar plaques 51. In animal models, this lipid metabolism dependent regulation significantly reduces Aβ plaque-induced neurite dystrophy and pathology-related neurotoxicity 51.

The R251G mutation occurs on the APOE *ε4* allele and is a rare missense mutation in which arginine is replaced by glycine at position 251(Figure 1). Studies have confirmed that this mutation reduces the risk of developing AD by 56%, and participants carrying this mutation show a significantly slower increase in the cumulative incidence of AD with age compared to non-carriers 53. The APOE4 (R251G) variant may mitigate the pathogenic risk of APOE4 by affecting the lipid-binding domain 53.

The APOE-R145C mutation is located within the receptor-binding region of APOE and is situated precisely between the two key variants (rs7412, i.e., R158C; rs429358, i.e., C112R) that determine the *ε2* and *ε4* alleles (Figure 1). This mutation is relatively common in individuals of African ancestry. Its association with the risk of AD is significantly genotype-dependent. The R145C mutation is significantly associated with an increased risk of AD and an earlier age of onset only in individuals with the APOE *ε3/ε4* genotype 54. No significant association was found in individuals with the APOE *ε2/ε3* or *ε3/ε3* genotypes 54. R145C carriers have nearly three times the risk of developing AD compared to non-carriers, and the reported age of onset is nearly six years earlier. Moreover, with increasing age, the cumulative incidence of AD in R145C carriers increases faster than in non-carriers 55.

## 3. Lipid metabolism and Membrane Organization in Synaptic Homeostasis

The critical role of lipid metabolism in AD has been recognized since Alois Alzheimer first observed lipid-rich inclusions in affected brains. In his neuropathological studies of AD patients, Alois Alzheimer first reported the abnormal presence of 'adipose inclusions' and 'lipoid granules' (lipid-like inclusions and lipid particles) in brain tissue 56. These pathological features have since been confirmed as structural signs of disrupted cerebral lipid homeostasis. The human brain is the second most lipid-rich organ after adipose tissue. Lipids make up approximately 10% to 12% of its fresh weight and over 50% of its dry weight. Recent research shows that in AD, genes involved in lipid processing and innate immunity form a statistically significant category of genetic risk factors, standing alongside the more classic categories of genes related to amyloid and tau processing 57. APOE, CLU, ABCA7, SORL1, and potentially TREM2 genes, which are the genetic variations linked to the risk of sporadic AD, are reported to be involved in lipid metabolism regulation 58. Among these, cholesterol and the ganglioside monosialotetrahexosylganglioside (GM1) have emerged as particularly important players, playing vital physiological roles in neuronal membranes and contributing to pathology when their homeostasis is disrupted 59.

Increasing evidence suggests that impairments in APOE-mediated lipid transport, particularly those associated with the APOE4 isoform, may compromise the ability of neurons to sustain the high level of synaptic turnover. Such disturbances in lipid homeostasis can influence the processing of amyloid precursor protein (APP), potentially shifting the balance between non-amyloidogenic and amyloidogenic pathways 60. In particular, reduced membrane cholesterol has been reported to favor non-amyloidogenic APP cleavage mediated by α-secretase (ADAM10) 14,15, whereas cholesterol-rich membrane microdomains are more permissive for β-secretase-mediated processing and Aβ generation 13. A growing number of human, animal, and cellular studies 61-71 have demonstrated that isoform-specific effects on lipid transport influence Aβ accumulation, synaptic integrity, and neuronal vulnerability, highlighting the importance of lipidation-dependent mechanisms in modulating AD risk.

### 3.1 Cholesterol Homeostasis and APP Processing

Cholesterol is a key sterol lipid that plays multiple essential roles in brain function, including maintaining synaptic plasticity, membrane stability, and cell signaling pathways 72. In the central nervous system, cholesterol mainly exists in its unesterified form and is primarily distributed within myelin sheaths and the membranes of neurons and glial cells 73. The brain's cholesterol metabolism is unique because the blood-brain barrier (BBB) restricts cholesterol entry from the bloodstream, requiring the brain to synthesize cholesterol locally rather than relying on peripheral cholesterol uptake 74. This localized production creates a specialized cholesterol homeostasis system within the CNS.

The cellular distribution of cholesterol synthesis in the brain follows a hierarchical organization. Astrocytes are the primary cholesterol producers in the adult brain, synthesizing and packaging cholesterol into apolipoprotein-containing lipoprotein particles for delivery to neurons 12. This astrocyte-to-neuron cholesterol transport system represents a critical metabolic interaction between these cell types 75. Microglia, the resident immune cells of the brain, also possess the capability to synthesize cholesterol, especially during stress responses or injury conditions, providing an additional cholesterol source. Mature neurons, while downregulating their cholesterol synthesis after embryogenesis under normal conditions, retain the ability to reactivate this pathway under pathological circumstances, suggesting an adaptive mechanism for maintaining local cholesterol homeostasis.

These mechanisms are most relevant for the axons of neurons with the greatest involvement in neuroplasticity, specifically, the long-range cortical projections from brainstem nuclei (locus coeruleus, raphe, nucleus basalis of Meynert) 76,77. Due to the massive scale and constant remodeling of these axonal arbors, their metabolic demands are exceptional. While CNS neurons produce enough cholesterol to survive and grow, the formation and maintenance of this vast number of mature synapses demands additional amounts of cholesterol provided by glia via APOE-containing lipoproteins 12.

While cholesterol metabolism has been implicated in AD through various mechanisms 78, one important role of APOE4 in AD pathogenesis may involve its impaired ability to regulate neuronal membrane lipid composition, which is required for membrane remodeling and synaptic integrity 79. Among the first structures affected in AD, the locus coeruleus (LC), raphe nuclei, and the nucleus basalis of Meynert exhibit exceptionally high synaptic turnover and thus have the greatest demands for efficient APOE-mediated cholesterol delivery. Lowering membrane cholesterol has been shown to enhance α-secretase activity, including ADAM10 80, likely by increasing membrane fluidity and improving enzyme access to APP, while also increasing the generation of the neuroprotective fragment sAPPα. Conversely, cholesterol-rich membrane environments are associated with greater amyloidogenic processing 13,81, in part because they favor β-secretase localization and activity. APOE4-associated defects in lipid handling may therefore bias APP processing toward a more amyloidogenic state, although the precise sequence of events remains to be fully established.

Beyond direct effects on ADAM10 activity, APOE-mediated cholesterol delivery also influences APP processing through membrane organization. BACE1 (β-secretase) is enriched in cholesterol-rich lipid rafts 13,82, and elevated cholesterol can enhance β-secretase cleavage 13,81, whereas cholesterol depletion suppresses this processing 83, potentially by altering the microenvironment required for BACE1 activity or its endocytic trafficking. γ-secretase is likewise highly dependent on cholesterol-rich membrane microdomains, and experimental cholesterol depletion has been reported to markedly reduce its activity, with restoration after cholesterol repletion. This APP partitioning into lipid rafts is the critical mechanistic step: once APP is localized to these cholesterol-enriched domains, it becomes more accessible to β- and γ-secretases that are also concentrated in these rafts, thereby promoting amyloidogenic processing 82 (Figure 4). Critically, lipid raft thickness serves as a key determinant of exactly where APP is cleaved by gamma-secretase, thereby shifting the balance between Aβ40 and the highly toxic Aβ42 84. The ratio of Aβ40 to Aβ42 is a critical issue, as these peptides may exert differential and potentially complementary effects on synaptic toxicity and aggregation propensity 85. In APOE4 carriers, altered membrane lipid organization may shift APP processing in ways that favor amyloidogenic cleavage and change the relative production of Aβ species and APP intracellular fragments, including AICD (APP-intracellular domain).

This framework may help explain why these neurons are affected early in AD: their exceptional plasticity requirements make them uniquely vulnerable to APOE4-associated alterations in cholesterol trafficking 79,86. Subsequently, the overt Alzheimer pathology, including both Aβ accumulation and phosphorylated tau (pTau), is linked to properly regulate APP processing 87.

### 3.2 GM1-Mediated Modulation of Aβ Aggregation

GM1 is a glycosphingolipid containing sialic acid and is one of the major gangliosides found in the nervous system. It is particularly concentrated in specialized membrane microdomains known as lipid rafts. These rafts serve as critical platforms for cellular signaling, protein trafficking, and membrane organization. The structural organization of GM1 is highly specific, which is anchored to the cell surface by inserting its lipid chain into the plasma membrane bilayer, while positioning its complex glycan portion on the outer layer of the cell membrane to interact with extracellular molecules 88.

The biosynthesis of GM1 follows a tightly regulated pathway beginning in the endoplasmic reticulum (ER) and continuing through the Golgi apparatus. Initially, ceramide (Cer) is synthesized in the ER and then transported to the Golgi, where a series of membrane-bound glycosyltransferases (GTs) sequentially add specific sugar residues to form the mature GM1 molecule 89. The cellular GM1 levels are maintained through a balance of synthesis and degradation, with degradation occurring primarily in lysosomes through the coordinated action of multiple hydrolytic enzymes including neuraminidases (Neu-ases), β-galactosidase (βGal-ase), β-hexosaminidase (βHex-ase), glucocerebrosidase (GCase), and ceramidase 90.

Under normal physiological conditions, GM1 plays vital roles in neurodevelopment and maintenance. It interacts with various cell surface receptors to promote key processes such as neuronal migration, dendrite formation, and axon growth. GM1 is especially important during neonatal cortical development, where it promotes neurite outgrowth in both peripheral and central neurons 91. However, in AD, GM1 assumes a more malignant role in disease pathogenesis. Research has shown that GM1 can directly bind to monomeric Aβ peptides, causing conformational changes that promote their aggregation into toxic soluble oligomers and downstream fibrils 59,92,93. This interaction appears to be highly specific. GM1 clusters in membrane microdomains form unique binding sites that stabilize particular Aβ conformations favorable for aggregation. Recent work by Zhang *et al*. has provided important mechanistic insights, showing that GM1 not only accelerates the formation of Aβ fibrils but also plays a critical role in stabilizing intermediate oligomeric species 94. This finding is particularly significant given that these soluble oligomeric forms of Aβ are increasingly recognized as the primary neurotoxic agents in AD pathogenesis.

GM1 has also been found to be present in secreted exosomes 95,96, and exosomes have also been shown to carry APOE 97. Studies have found that the GM1 level in detergent-resistant membrane microdomains of synaptosomes is significantly higher in APOE4 knock-in mouse brains compared to those with APOE3 98. Zhang *et al*. showed that APOE regulates the intercellular transport of GM1, and this process is modulated by the expression levels of APOE receptors 99. This discovery reveals a novel functional link between GM1 and APOE in AD pathogenesis, suggesting their potential interplay in modulating synaptic membrane organization and Aβ aggregation dynamics.

GM1's pathological role in AD is a clear example demonstrating that even membrane components beneficial under normal conditions can turn harmful under disease conditions. Together with the abnormal cholesterol metabolism, changes in GM1 dynamics create a lipid environment that actively promotes Aβ aggregation and toxicity. This finding identifies lipid metabolism as a central part of AD pathogenesis and suggests that therapeutic strategies targeting lipid homeostasis might offer promising approaches for disease intervention.

## 4. Structural Dynamics, Lipidation, and Receptor Interactions of APOE

APOE is a multifunctional protein critical for many cellular processes such as lipid transport, synaptic maintenance, and immune regulation. To perform these diverse roles, APOE undergoes a tightly regulated biogenesis and maturation process 100-103. While APOE is synthesized and secreted by glial cells, its functional assembly into lipoprotein particles depends on lipidation mediated by ATP-binding cassette (ABC) transporters. Specifically, ABCA1 is essential for the initial lipidation of nascent, lipid-poor APOE to form discoidal particles 102. Subsequently, ABCG1 facilitates the further expansion of these particles by promoting additional cholesterol efflux 100. These mature, spherical lipoproteins then circulate within the brain parenchyma and cerebrospinal fluid (CSF) to maintain lipid homeostasis 104. Although the exact details of this maturation process are not fully understood, it likely involves the transfer of additional lipids, such as cholesteryl esters and phospholipids 105. Through these steps, APOE is converted into high-density lipoprotein (HDL)-like particles. Lipidation not only stabilizes APOE structurally but also exposes functional domains necessary for receptor binding and efficient lipid transport, making this process indispensable for maintaining lipid homeostasis in both the central nervous system (CNS) and periphery. The following sections will explore the structural and functional consequences of this process, beginning with the characteristics and significance of the lipid-free APOE state.

### 4.1 Lipid-Free APOE

Following its secretion from cells, APOE initially exists in a lipid-free state before undergoing lipidation through interactions with lipid transporters. Lipid-free APOE in solution tends to self-assemble into aggregates *in vitro* in an isoform-dependent manner under near-physiological conditions, and this aggregation is effectively impeded by the lipidation of APOE 106. APOE4 shows the highest propensity for aggregation, followed by APOE3 and APOE2. This aggregation occurs through a stepwise process, beginning with the formation of dimers and tetramers, which can further assemble into higher-order structures, including fibrils 67. The R61T mutation increases hinge region flexibility and disrupts domain-domain interaction of APOE4 107. APOE4, which is less efficiently lipidated compared to APOE2 and APOE3, forms larger and more stable aggregates that are particularly toxic to neuronal cells. These aggregates disrupt cellular function and contribute to the pathological processes observed in neurodegenerative diseases such as AD 108. The aggregation of lipid-free APOE is not merely a structural issue but also has functional consequences. Unlipidated APOE isoforms, particularly APOE4, are less effective in binding to lipids and cellular receptors, impairing their ability to mediate cholesterol transport and Aβ clearance 109. This deficiency underscores the importance of lipidation in maintaining APOE functional integrity and preventing its pathological aggregation.

### 4.2 Lipidation Process

Following its initial secretion in a lipid-free state, APOE undergoes a critical lipidation process that fundamentally transforms its structure and function (Figure 1). Lipidation is a dynamic process that involves the binding of lipids, such as cholesterol, phospholipids, and triglycerides, to APOE. The C-terminal domain of APOE is crucial in this process, as lipid binding induces a reorientation of the α-helices within this domain, positioning them perpendicular to the acyl chains of the lipids and thereby defining the periphery of the nanodisks 110 (Figure 1). The lipidation state of APOE influences its structural conformation, receptor-binding capacity, and functional properties. Upon lipid association, APOE undergoes structural rearrangements that expose functional domains. Helix 4 of the N-terminal bundle, enriched in positively charged lysine and arginine residues (residues 136-150), serves as the primary receptor-binding region 111 (Figure 1). Lipid binding destabilizes the salt bridges tethering the N- and C-terminal domains, leading to a "hinge-opening" motion that undocks the receptor-binding region from its buried position 112. This conformational shift enhances accessibility to LDL receptor family members (e.g., LRP1), enabling efficient lipid delivery. While models differ on the extent of N-terminal domain reorganization, consensus suggests that lipid-bound APOE adopts an extended conformation, optimizing both lipid transport and receptor engagement.

APOE isoforms display distinct lipidation patterns due to variations in their amino acid sequences. For instance, APOE2 and APOE3 preferentially bind to small, phospholipid-rich HDL particles, which are highly efficient in cholesterol transport and Aβ clearance 113. APOE2, which undergoes extensive lipidation, forms larger lipoprotein complexes (480-720 kDa in the CSF) and exhibits superior cholesterol efflux capacity compared to APOE3 114. In contrast, APOE4 shows a stronger affinity for larger, triglyceride-rich very low-density lipoprotein (VLDL) particles, which are less effective in these functions 115. Poorly lipidated APOE4 is associated with smaller lipoprotein complexes (<500 kDa in the CSF), reduced cholesterol content, and an elevated risk of AD. Furthermore, compared to APOE2 and APOE3, APOE4 is linked to a significant reduction in ten major lipid classes, including phosphatidylethanolamine, phosphatidic acid, and mitochondrial membrane bilayer-forming phospholipids 115. This differential binding affinity results in a higher proportion of unlipidated APOE4, which promotes its aggregation and contributes to neurotoxic effects.

In the periphery, APOE4 is associated with elevated LDL-cholesterol levels, while APOE2 is linked to lower cholesterol levels but higher triglyceride levels 116. These differences in lipid metabolism highlight the complex interplay between APOE isoforms, lipidation states, and disease risk.

### 4.3 Lipidated APOE and Receptor Interactions

**Figure 2:**
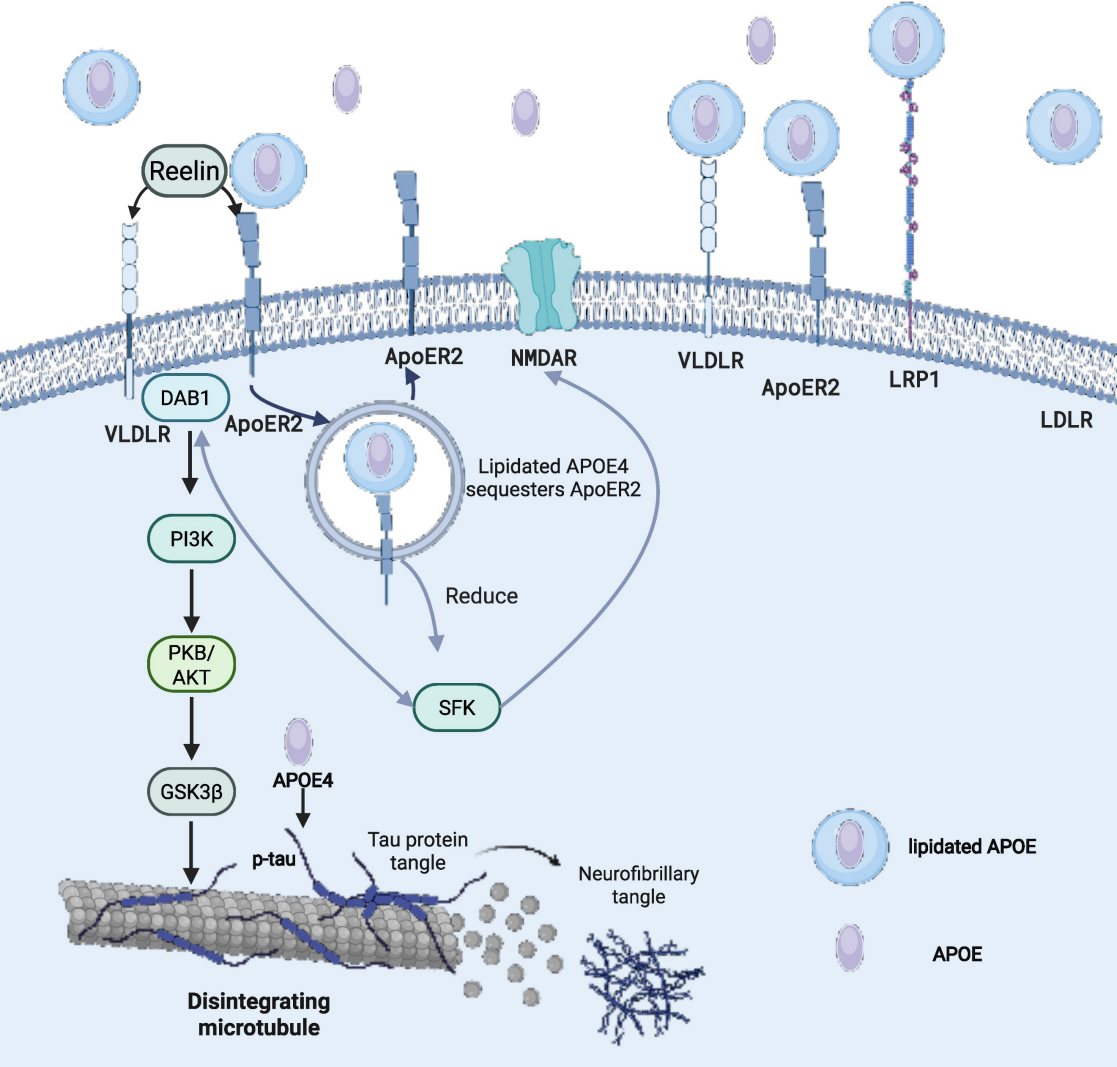
** Lipidated APOE-receptor interactions regulating lipid transport, Reelin signaling, and tau phosphorylation in the CNS.** Lipidated APOE particles engage LDL receptor family members - including LDLR, LRP1, VLDLR, and APOER2 - to mediate cholesterol and lipid redistribution from astrocytes to neurons. LDLR and LRP1 drive APOE lipoprotein clearance in glia and neurons, respectively, whereas VLDLR and ApoER2 transduce Reelin signals via Dab1-SFK-PI3K-AKT to inhibit GSK3β and restrain tau phosphorylation. In AD, APOE4-containing lipoproteins aberrantly sequester APOER2 and glutamate receptors (NMDAR/AMPAR) intracellularly, impairing Reelin-enhanced synaptic function and promoting GSK3β-dependent tau hyperphosphorylation, microtubule disintegration, and neurofibrillary tangle formation. Created with BioRender.com.

Lipidated APOE molecules play a crucial role in various biological processes, including triggering the uptake of APOE lipoprotein or activating downstream signaling pathways 117. In the CNS, the clearance of APOE lipoprotein relies on the uptake process that is mediated by APOE receptors 118. When APOE binds to lipids, it undergoes a conformational change that allows it to interact with members of the LDL receptor family, such as LDLR, LRP1, very low-density lipoprotein receptor (VLDLR), and apolipoprotein E receptor 2 (APOER2) (Figure 1). Among them, LDLR and LRP1 are the primary receptors responsible for APOE binding to lipoproteins and lipid delivery. In the CSF, no other ligands for LDLR and LRP1 have been identified besides APOE lipoproteins. LDLR is widely expressed in glial cells, whereas LRP1 is mainly found in neurons 119. Notably, LDLR only binds to lipidated APOE 120. The absence of LDLR and LRP1 results in elevated APOE levels in the mouse brain 121,122. VLDLR and APOER2 also function as key components of the Reelin signaling pathway, which regulates neurodevelopmental processes such as synaptic plasticity, dendritic arborization, neuronal migration patterning, and cognitive function regulation 123. When Reelin binds to VLDLR and APOER2, it triggers the intracellular signaling cascade, beginning with the phosphorylation of adapter protein Dab1 by Src family tyrosine kinases (SFK), then activates a kinase cascade involving phosphatidylinositol-3-kinase (PI3K) and protein kinase B (PKB/Akt), while also inhibiting glycogen synthase kinase 3β (GSK3β), which is one of the major kinases involved in tau phosphorylation 124 (Figure 2).

The presence of cysteine at position 158 in APOE2 changes the conformation of the positively charged receptor-binding domain, reducing its affinity for the LDLR 125 (Figure 1). This weakened binding of APOE2 to LDLR causes the accumulation of triglyceride-rich lipoprotein remnants, which contributes to the onset of Type III hyperlipoproteinemia in some individuals homozygous for APOE2 126.

Through their interaction with lipidated APOE, APOE receptors mediate the transfer of cholesterol and other key lipids from astrocytes to neurons. This directional transport is essential for maintaining fundamental neuronal functions, including cellular growth, membrane homeostasis, and synaptogenesis. APOE3 plays role in promoting neurite outgrowth and protecting neurons from programmed cell death, which depends on LRP1 127.

One of the earliest pathological features of AD is synaptic dysfunction 128. Lipidated APOE4 derived from astrocytes traps APOER2, N-methyl-D-aspartate receptor (NMDAR), and α-amino-3-hydroxy-5-methyl-4-isoxazolepropionic acid receptor (AMPAR) within intracellular compartments, leading to their reduced expression (Figure 2). This sequestration impairs Reelin's ability to enhance synaptic glutamate receptor activity 129. Notably, this pathological reduction in receptor availability affects normal synaptic plasticity rather than the pathological overactivation targeted by NMDA receptor antagonists such as memantine. Lipidated APOE2 enhances the rate of synapse pruning and turnover in the brain by astrocytes 60.

## 5. Modulation of the Aβ Lifecycle by APOE Isoforms and Lipidation

Aβ is not a static pathological byproduct, but a dynamically regulated peptide undergoing continuous production and clearance in the central nervous system. Stable isotope labeling kinetics studies in humans demonstrate that Aβ is rapidly turned over under physiological conditions, with synthesis and clearance maintained in a tightly coupled equilibrium 130. Failure to maintain this homeostasis can stem from different primary defects. For instance, in Down syndrome, a lifelong 50% excess of the APP gene (due to Trisomy 21) drives a continuous, primary overproduction of Aβ, leading to early-onset accumulation 131. In contrast, studies in patients with symptomatic, sporadic AD indicate that Aβ accumulation is more commonly associated with impaired clearance rather than increased production 132. These findings highlight that APP processing and Aβ generation must be interpreted within a broad framework that integrates both production and clearance dynamics.

### 5.1 Regulation of APP Processing and Amyloidogenic Cleavage

Membrane lipid composition represents a critical proximal regulator of APP processing. Cholesterol-rich membrane microdomains (lipid rafts) are particularly important for amyloidogenic processing. Experimental depletion of neuronal cholesterol markedly suppresses Aβ generation while preserving non-amyloidogenic APP processing, indicating that cholesterol is required for β-secretase-mediated cleavage 133. These observations suggest that the lipid environment of neuronal membranes affects whether APP is preferentially processed via α- or β-secretase pathways. Given that APOE is the primary lipid transport protein in the brain, its isoform-specific properties and lipidation status are expected to play a central role in modulating membrane composition, APP trafficking, and Aβ production.

Consistent with this view, APP itself plays an essential role in maintaining synaptic structure and function through its non-amyloidogenic processing pathway. The α-secretase-derived fragment APPsα has been shown to be a key regulator of synaptic plasticity. Conditional deletion of APP and APLP2 in the adult brain leads to impaired dendritic architecture, reduced spine density, and severe deficits in long-term potentiation and memory, all of which can be rescued by acute supplementation of APPsα 134. Similarly, expression of APPsα alone is sufficient to restore anatomical, behavioral, and electrophysiological abnormalities in APP-deficient mice 135. These findings emphasize that shifts in APP processing not only increase Aβ production but also reduce the availability of neuroprotective APPsα, thereby disrupting synaptic homeostasis.

Emerging evidence indicates that APOE isoforms directly influence APP processing and Aβ generation through multiple mechanisms. In neuronal cell models, lipid-poor ApoE4 induces significantly higher Aβ production than ApoE3, an effect that depends on low-density lipoprotein receptor-related protein (LRP)-mediated endocytosis and enhanced recycling of APP to endosomal compartments where amyloidogenic processing occurs 136 (Figure 4). Notably, this effect is linked to the unique domain interaction within ApoE4, as disruption of this structural feature attenuates its ability to promote Aβ production.

In addition to regulating APP trafficking, ApoE also functions as a signaling molecule that directly modulates APP expression. ApoE binding to its neuronal receptors activates a non-canonical MAP kinase signaling cascade involving DLK, MKK7, and ERK1/2, leading to AP-1-dependent transcriptional upregulation of APP and increased Aβ secretion 137 (Figure 4). Importantly, the potency of this effect follows the order ApoE4 > ApoE3 > ApoE2, mirroring their relative risk for AD. This mechanism demonstrates that APOE isoforms regulate Aβ production not only at the level of APP processing but also at the level of APP gene expression.

Systemic lipid metabolism may further modulate this axis. Dietary intake of trans fatty acids significantly alters circulating lipoprotein profiles by increasing LDL and decreasing HDL levels 138. Although the relationship between peripheral lipid metabolism and brain cholesterol homeostasis is complex, these findings suggest that systemic lipid states could influence APOE lipidation and membrane composition. Consistent with this notion, epidemiological studies have reported associations between statin use and reduced AD risk, particularly among APOE ε4 carriers, although these findings remain correlative and require further validation 139,140.

Furthermore, ApoE4 also disrupts ABCA1 function, creating a pathogenic feedback loop. In astrocytes, ApoE4 impairs ABCA1 recycling to the plasma membrane through ARF6-dependent trafficking defects, reducing cholesterol efflux and ApoE lipidation 141. This results in the accumulation of poorly lipidated ApoE particles. Consequently, ApoE4 indirectly drives more amyloidogenic processing while simultaneously amplifying its pathogenic impact.

In summary, APOE isoforms and their lipidation states regulate APP processing and Aβ production through a multifaceted network involving membrane lipid composition, receptor-mediated trafficking, intracellular signaling, and protein-protein interactions. Rather than acting at a single step, ApoE4 and impaired lipidation shift the entire system toward increased amyloidogenic processing and reduced homeostatic buffering.

### 5.2 Receptor-Mediated Trajectories of Aβ Clearance and Efflux

#### Blood-Brain Barrier Transcytosis and Cellular Uptake Mechanisms

Aβ clearance from the brain is a highly coordinated, multi-step process involving vascular transport, cellular uptake, intracellular trafficking, and enzymatic degradation. Early work established that Aβ is removed primarily through two major pathways: transcytosis across the blood-brain barrier (BBB) and bulk flow along the interstitial fluid (ISF), with BBB-mediated clearance being substantially more efficient. In particular, Aβ40 is rapidly cleared via LRP1-dependent transport across the BBB, whereas Aβ42, which is more aggregation-prone, is cleared more slowly. Importantly, the formation of Aβ-apoE complexes significantly reduces BBB clearance efficiency, suggesting that APOE can directly modulate Aβ retention within the brain 142.

A key determinant of Aβ clearance efficiency is the APOE isoform. In vivo studies using microdialysis and stable isotope labeling have demonstrated that Aβ production rates are largely unchanged across APOE genotypes, whereas clearance rates differ markedly, following the order ApoE2 > ApoE3 > ApoE4. Notably, this impairment in Aβ clearance is evident prior to plaque deposition, indicating that defective clearance is an early and driving event in AD pathogenesis 143. Mechanistically, APOE isoforms influence receptor usage: ApoE4 redirects Aβ-apoE complexes away from the fast LRP1-mediated pathway toward the slower VLDLR pathway, effectively trapping Aβ in a less efficient clearance route. In contrast, ApoE2 and ApoE3 retain partial access to LRP1-mediated transport, resulting in more efficient removal 16 (Figure 5).

At the level of the neurovascular unit (NVU), Aβ clearance is mediated by the coordinated action of multiple cell types and transport proteins. In endothelial cells, LRP1 functions as the primary receptor for Aβ uptake from the brain side, PICALM regulates vesicular trafficking and endocytic sorting, and ABCB1/P-gp mediates the final efflux of Aβ into the circulation. Together, these components form an integrated transcytotic system that governs BBB clearance of Aβ 144 (Figure 5). Beyond endothelial cells, pericytes, astrocytes, and microglia contribute to Aβ clearance through complementary mechanisms. Pericytes internalize Aβ via LRP1, astrocytes and microglia produce APOE and facilitate uptake and degradation, and ABCA1-dependent lipidation of APOE is essential for maintaining its functional capacity within this system 145.

**Figure 3:**
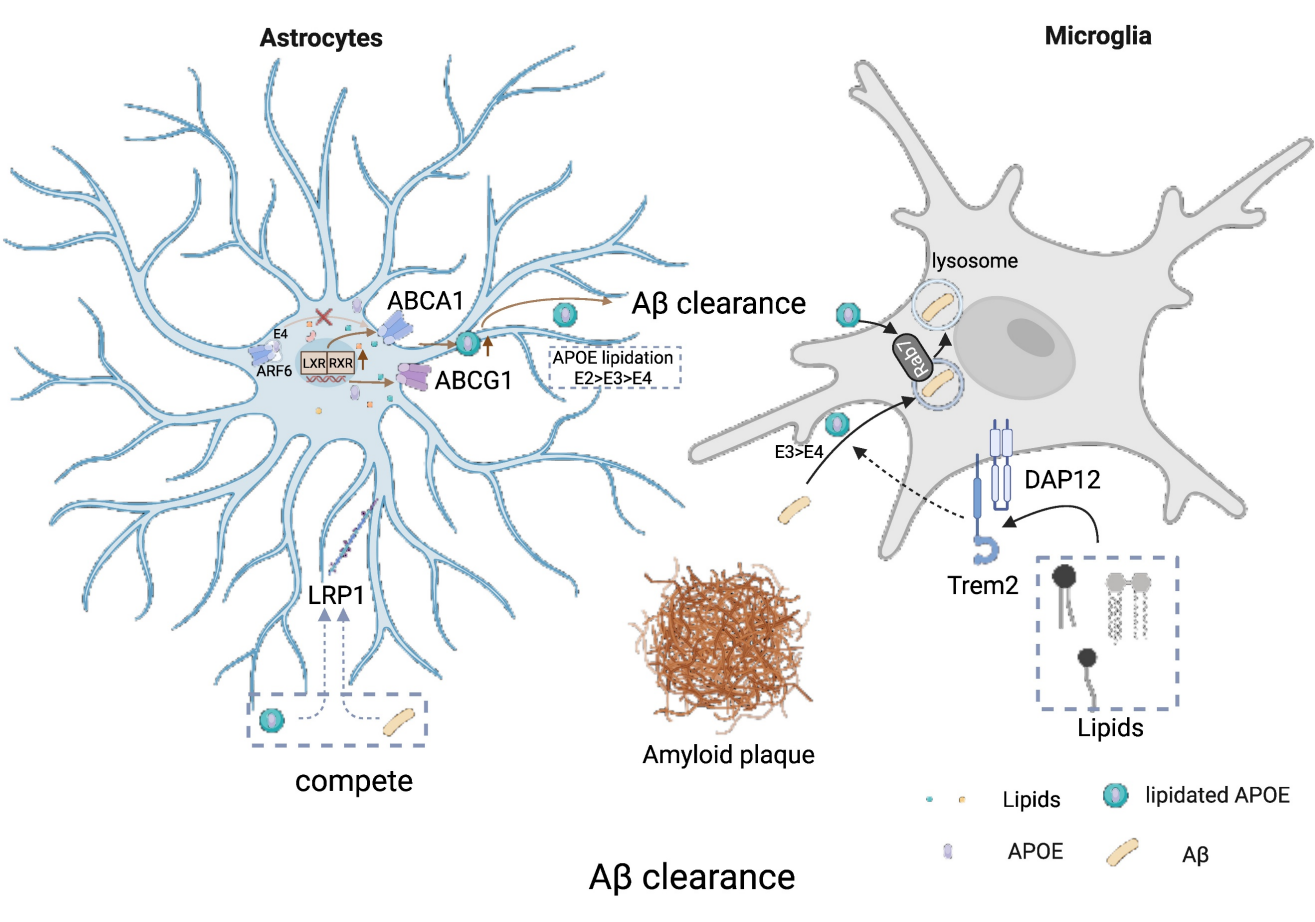
** APOE isoform- and lipidation-dependent regulation of Aβ clearance by astrocytes and microglia.** Astrocyte ABCA1- and ABCG1-mediated lipid efflux controls the lipidation state of secreted APOE, which in turn affects its ability to bind and clear Aβ. Well-lipidated APOE (E2 > E3 > E4) more effectively solubilizes Aβ and promotes its clearance via LRP1-mediated uptake, whereas poorly lipidated APOE4 favors Aβ aggregation and plaque formation. In microglia, lipidated APOE particles are internalized through TREM2-DAP12 signaling and delivered to lysosomes for degradation of Aβ. APOE4's impaired lipidation, due in part to ARF6-dependent mistrafficking of ABCA1, reduces both astrocytic and microglial Aβ clearance, accelerating amyloid pathology. Pharmacological activation of LXR/RXR enhances ABCA1 expression, boosts APOE lipidation, and ameliorates Aβ burden in AD models, highlighting lipidation as a therapeutic target. Created with BioRender.com.

**Figure 4:**
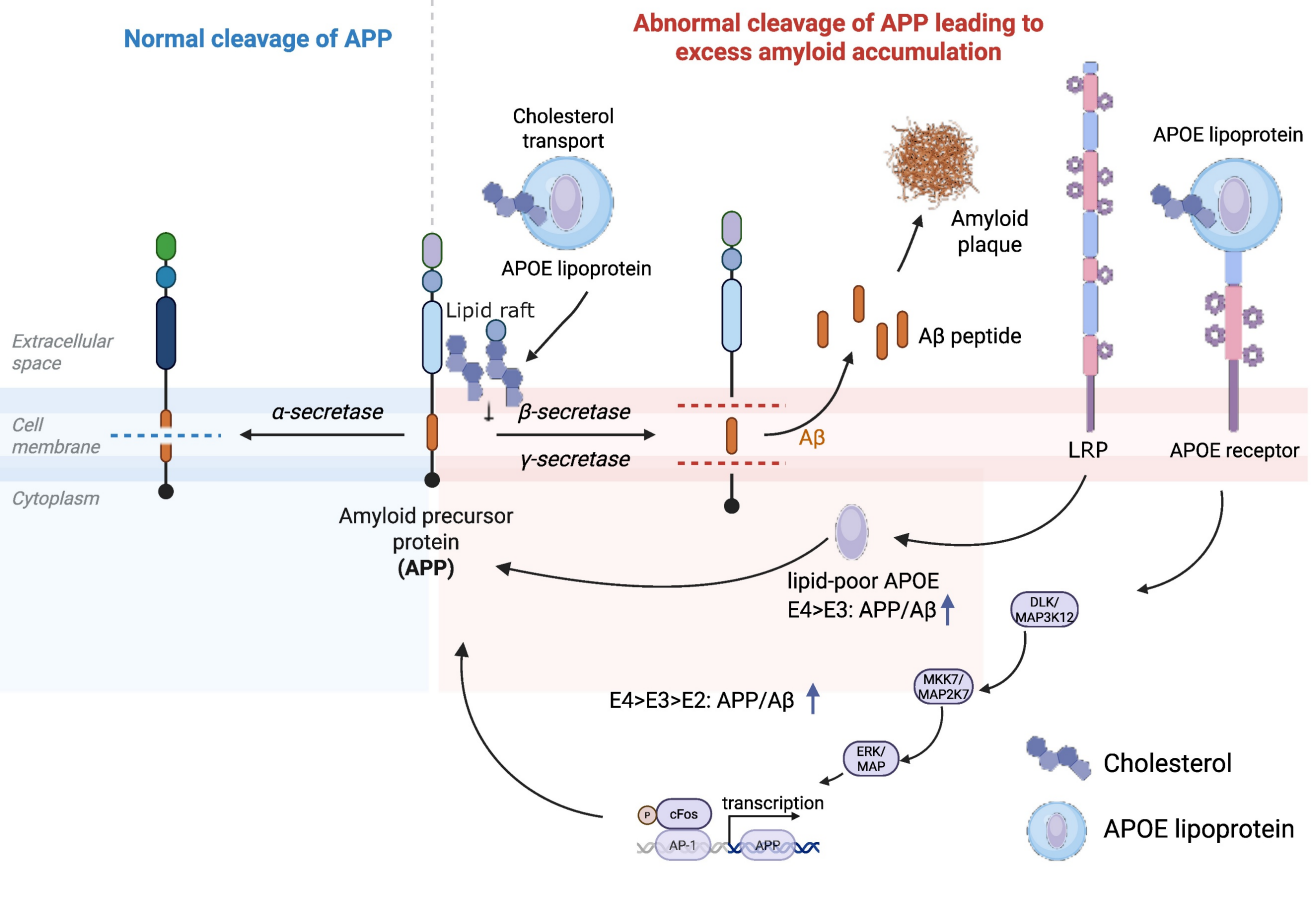
** APOE isoform- and lipidation-dependent regulation of APP processing and Aβ generation via LRP-mediated uptake and DLK/MAPK signaling.** Lipid-poor APOE4 enhances β-secretase cleavage of APP and Aβ production more strongly than APOE3 through two complementary mechanisms. First, APOE4 interaction with LRP promotes APP endocytosis into β/γ-secretase-containing compartments, an effect blocked by receptor-associated protein or LRP knockdown and abolished by disrupting APOE4 domain interaction. Second, APOE binding to its receptors activates DLK→MKK7→ERK1/2 kinase cascade, leading to c-Fos/AP-1-driven upregulation of APP transcription and further increasing Aβ generation. APOE lipidation state the cholesterol-mediated distribution of APP into microdomains further tune these processes, where poorly lipidated APOE4 promotes the co-localization of APP with secretases within lipid rafts, driving the greatest amyloidogenic APP processing. Created with BioRender.com.

At the cellular level, APOE isoforms differentially regulate Aβ uptake, trafficking, and degradation. Pericytes rely on LRP1 to internalize aggregated Aβ42, a process that is supported by ApoE3 but impaired by ApoE4, leading to reduced clearance efficiency 146. Within neurons and glial cells, internalized Aβ is trafficked from early endosomes (Rab5-positive) to late endosomes (Rab7-positive) and ultimately to lysosomes for degradation (Figure 3), although a fraction can be recycled back to the extracellular space via Rab11-positive compartments. ApoE3 promotes Aβ uptake and efficient lysosomal targeting, whereas ApoE4 is less effective in directing Aβ into degradative pathways, resulting in intracellular retention and increased toxicity 147. Consistently, human iPSC-derived models show that APOE4 impairs astrocytic Aβ clearance, reduces microglial phagocytic capacity, and increases neuronal Aβ42 production, collectively creating a cellular environment that favors Aβ accumulation 148. In parallel, sleep-dependent clearance mechanisms play a critical role in maintaining Aβ homeostasis, and disruption of sleep exacerbates Aβ accumulation, particularly in the presence of APOE4 24,149.

#### Competitive Receptor Binding and Enzymatic Degradation

**Figure 5:**
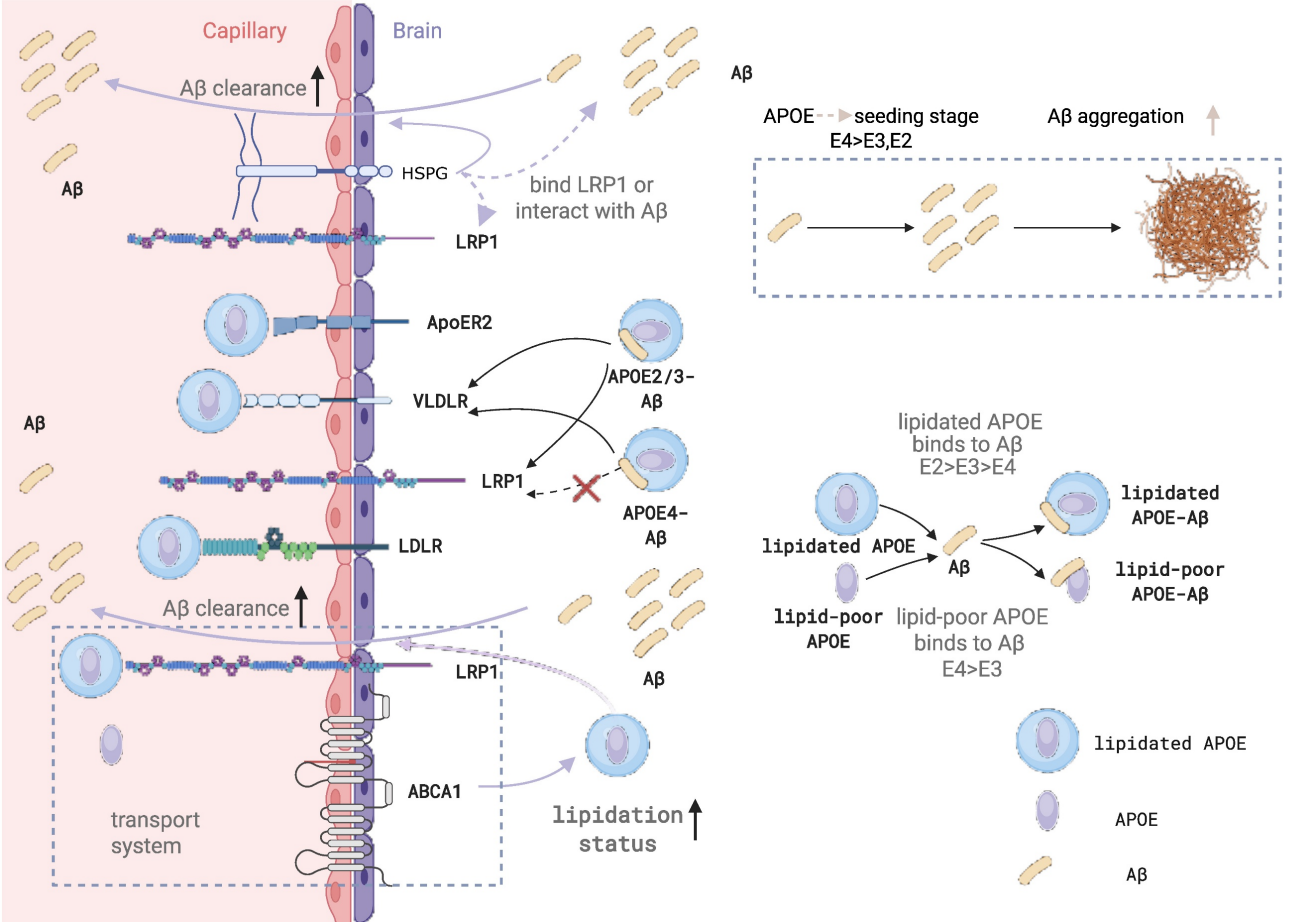
** APOE isoform and lipidation influence on Aβ efflux pathways through the BBB.** Lipidation status of APOE significantly alters its interaction with Aβ and subsequent transport across the BBB. Lipidated APOE binds Aβ with higher affinity than lipid-poor APOE, with isoform-dependent differences (E2 > E3 > E4). While lipidated APOE3 can inhibit Aβ40 transcytosis across the BBB, lipid-poor APOE3 partially impairs it. APOE4-bound Aβ is preferentially cleared via the slower VLDLR pathway, whereas APOE2 and APOE3 complexes utilize both LRP1 and VLDLR for more efficient transport. Additionally, ABCA1-mediated APOE lipidation at the abluminal side of the BBB enhances APOE-Aβ complex formation and promotes clearance through the LRP1/ABCB1 transport system. HSPGs further support Aβ clearance by facilitating interactions with LRP1 or directly binding Aβ, thereby limiting aggregation.

While APOE can form complexes with Aβ and influence its transport and clearance, its effects are not solely dependent on direct binding. Under physiological conditions, most Aβ exists in a free, unbound state, suggesting that additional mechanisms contribute to APOE-mediated regulation of Aβ clearance 27. One such mechanism is a competition-based model in which APOE and Aβ share common clearance pathways, particularly those mediated by receptors such as LRP1 (Figure 3). In this context, APOE, especially ApoE4, can compete with Aβ for receptor binding and intracellular trafficking machinery, thereby limiting Aβ access to efficient clearance routes, further exacerbating Aβ retention in the brain. Specifically, lipidated APOE particles (both APOE3 and APOE4) have been shown to inhibit cellular Aβ uptake by competing for binding sites on cell-surface HSPGs 150. Beyond direct Aβ interactions, cell-surface HSPGs also play a critical role in Aβ clearance by facilitating the uptake of APOE-Aβ complexes or lipoprotein remnants (Figure 5). This clearance can occur either through the formation of an HSPG-LRP1 endocytic complex or via an HSPG-only sequestration pathway 151.

In addition to transport and cellular uptake, enzymatic degradation represents a critical component of Aβ clearance. Neprilysin (NEP) is a major rate-limiting enzyme responsible for degrading Aβ42 in the brain parenchyma, and its inhibition leads to rapid accumulation and deposition of Aβ 152. Insulin-degrading enzyme (IDE) also contributes to extracellular Aβ degradation at physiological concentrations and can be competitively inhibited by insulin, providing a mechanistic link between metabolic dysfunction and AD risk 153. Importantly, NEP expression and activity are reduced in AD brains, particularly in APOE4 carriers, suggesting that APOE genotype may also influence proteolytic clearance capacity 154.

A critical modulator of APOE function in Aβ clearance is its lipidation state. APOE lipidation, mediated by ABCA1, is essential for its structural integrity and functional activity (Figure 3). Lipidated APOE enhances cholesterol efflux and promotes efficient intracellular trafficking of Aβ toward lysosomal degradation by facilitating Rab7 recycling and endolysosomal transport 17. In astrocytes, APOE is required for the recognition and degradation of deposited Aβ, acting as a key regulator of plaque-associated clearance 18. Beyond its role in trafficking, APOE also directly facilitates the proteolytic degradation of Aβ 19. In microglia, APOE enhances NEP-mediated degradation of soluble Aβ 152, while in the extracellular space it promotes IDE-dependent proteolysis 153. These effects are strongly dependent on APOE lipidation, as ABCA1 deficiency leads to reduced APOE lipidation, impaired Aβ degradation, and increased amyloid burden. Furthermore, APOE isoforms differ in their ability to promote proteolysis, with ApoE3 being more effective than ApoE4 19.

Interestingly, APOE lipidation also introduces pathway-specific trade-offs. While lipidated APOE enhances cellular uptake and enzymatic degradation, lipidated APOE-Aβ complexes are cleared more slowly across the BBB compared to non-lipidated forms, suggesting that lipidation differentially modulates distinct clearance routes 16. Therapeutically, enhancing APOE lipidation via activation of the LXR-ABCA1 axis has shown promise: increasing APOE lipidation improves Aβ clearance, reduces amyloid burden, and ameliorates cognitive deficits in AD models.

Taken together, these findings support a unified model in which APOE isoforms and lipidation status coordinately regulate Aβ clearance across multiple interconnected processes, including vascular transport, receptor-mediated uptake, intracellular trafficking, and enzymatic degradation. ApoE2 and ApoE3 generally promote efficient clearance, whereas ApoE4 disrupts these processes at multiple levels, leading to a system-wide impairment of Aβ homeostasis. This integrative framework highlights APOE lipidation as a central regulatory node and a potential therapeutic target for restoring Aβ clearance in Alzheimer's disease.

### 5.3 Control of Aβ Nucleation and Aggregation Dynamics

#### Pathogenic Significance of Soluble Aβ Oligomers

Aβ production is closely linked to neuronal and synaptic activity. In vivo microdialysis studies have shown that increased neuronal firing rapidly elevates interstitial fluid Aβ levels, whereas inhibition of action potentials or synaptic vesicle release significantly reduces Aβ concentrations 155. This coupling suggests that Aβ is released as part of normal synaptic function rather than exclusively generated under pathological conditions. Importantly, Aβ exhibits a concentration-dependent dual effect: at picomolar levels, it enhances synaptic plasticity and memory, whereas at higher concentrations it impairs long-term potentiation and contributes to synaptic dysfunction 156.

Aβ, a central pathological hallmark of AD, exists in a dynamic equilibrium of multiple aggregation states, including monomers, soluble oligomers, protofibrils, fibrils, and plaques. These species differ substantially in their biological activity. Increasing evidence 3-7 suggests that soluble oligomeric Aβ species are major drivers of synaptic dysfunction and cognitive decline. Early studies demonstrated that fibril-free synthetic Aβ oligomers potently inhibit long-term potentiation (LTP) 3, indicating strong neurotoxicity even in the absence of plaque formation. Consistent with this concept, patients carrying the Osaka mutation (APP E693Δ) develop severe dementia accompanied by high levels of oligomeric Aβ despite minimal amyloid plaque deposition 4. Moreover, structural studies have further distinguished these toxic species, identifying that only specific high-molecular-weight oligomers possess the quaternary structure necessary to bind synapses and drive dysfunction 5,7. Furthermore, soluble Aβ oligomers can trigger complement deposition on synapses and promote microglia-mediated synaptic pruning, leading to early synapse loss prior to overt plaque formation 157. More recently, therapeutic agents such as Lecanemab, which preferentially target soluble protofibrils, have shown clinical benefits 6, further supporting the pathogenic importance of these metastable Aβ assemblies.

#### APOE-Dependent Initiation of Aβ Nucleation and Early Assembly

Crucially, Aβ clearance and Aβ aggregation must be understood as opposing outcomes that are fundamentally dictated by the lipidation state of APOE. While adequately lipidated APOE engages receptors to promote the successful clearance of soluble Aβ (as discussed in Section 5.2), lipid-poor or unlipidated APOE fails to mediate this removal and instead actively facilitates Aβ nucleation and deposition. Thus, APOE not only influences Aβ metabolism through failed clearance pathways, but also directly participates in Aβ aggregation in an isoform-dependent manner. Early neuropathological studies demonstrated that ApoE co-localizes with amyloid deposits in senile plaques and other amyloid lesions, suggesting that ApoE is not merely passively associated with pre-formed aggregates but may be involved in the aggregation process itself 21. This notion is further supported by genetic and postmortem studies showing that APOE genotype directly influences cerebral Aβ deposition, with APOE4 exhibiting a strong gene dose-dependent increase in plaque burden 41. Together, these findings support an active role for APOE in modulating Aβ aggregation.

Mechanistically, the interaction between APOE and Aβ is highly dependent on the conformational state of Aβ. Biochemical studies have shown that ApoE preferentially binds Aβ species adopting β-sheet structures, which correspond to aggregation-prone intermediates during the transition from monomers to oligomers and higher-order assemblies 22. This conformational selectivity suggests that APOE primarily acts at early stages of aggregation, where it can influence the trajectory of Aβ assembly by interacting with these intermediate species rather than simply altering total Aβ levels.

Isoform-specific differences in APOE further shape the distribution of Aβ species along the aggregation pathway. In APOE4 carriers, levels of soluble ApoE/Aβ complexes are reduced, while oligomeric Aβ species are increased, indicating that ApoE4 forms less stable complexes with Aβ that are more prone to dissociation 20. This instability likely increases the availability of free Aβ capable of self-assembly, thereby favoring oligomer formation. In this framework, APOE does not simply promote or inhibit aggregation globally, but rather regulates the partitioning of Aβ between soluble complexed states and aggregation-prone species.

#### Stage-Dependent Seeding Effects and the Unresolved Role of Lipidation

In vivo studies further indicate that APOE exerts a stage-dependent effect on Aβ aggregation, acting predominantly during the early seeding phase. Inducible expression models demonstrate that ApoE4 significantly accelerates the initiation of amyloid pathology when present during early aggregation (Figure 5), whereas its impact is markedly reduced once plaques are already established 69. Similarly, reducing ApoE levels prior to plaque formation suppresses subsequent Aβ deposition, whereas intervention after seeding has limited effects on overall plaque burden 158. These findings collectively support a model in which APOE primarily modulates the initial nucleation and early assembly of Aβ rather than later-stage fibril growth.

In contrast, the role of APOE lipidation in Aβ aggregation remains less well defined. Although lipidation profoundly affects ApoE structure and its ability to interact with Aβ, most existing studies have focused on its role in Aβ clearance and transport rather than its direct impact on aggregation kinetics. Available evidence suggests that differences in ApoE/Aβ complex stability, observed across APOE isoforms, may partly reflect differences in lipidation state; however, this relationship remains largely inferential and has not been directly established as a mechanism regulating Aβ aggregation 20.

However, the lipidation status of APOE is a key determinant of its function in Aβ metabolism. The ATP-binding cassette transporter ABCA1 is essential for loading lipids onto ApoE particles, thereby regulating their structural stability and biological activity. Deletion of ABCA1 in AD mouse models results in reduced ApoE levels but paradoxically increased Aβ deposition, indicating that poorly lipidated ApoE is more prone to promote Aβ aggregation and plaque formation 159. Conversely, overexpression of ABCA1 enhances ApoE lipidation and significantly reduces amyloid burden 160. These findings highlight that the functional “quality” of ApoE, particularly its lipidation state, is more critical than its absolute abundance.

The importance of lipidation is further supported by biophysical studies showing that ApoE-Aβ interactions are highly dependent on the lipid-bound state of ApoE. In its native lipidated form, ApoE3 exhibits stronger binding affinity to Aβ than ApoE4, whereas delipidation abolishes isoform-specific differences 161,162 (Figure 5). Additional studies demonstrate that ApoE4 binds Aβ more rapidly under certain conditions, suggesting that isoform-specific interactions are highly sensitive to conformational state and biochemical context 163. These results underscore that physiologically relevant interpretations of APOE function must consider its lipidation status.

Overall, current evidence supports a model in which APOE isoforms directly influence Aβ aggregation by modulating early conformational selection and seeding processes, thereby determining whether Aβ enters oligomerization pathways. Future studies are needed to better delineate how APOE lipidation independently regulates Aβ aggregation.

## 6. Modulation of Tau pathology by APOE Isoforms and Lipidation

### 6.1. Amyloid-Independent Pathways of Tau Vulnerability

A growing body of clinical and imaging evidence indicates that APOE influences tau pathology not merely through its established effects on Aβ, but also through mechanisms that are at least partly independent of Aβ burden. Tau PET studies have shown that APOE ε4 carriers exhibit increased tau accumulation in the medial temporal lobe, particularly in the entorhinal cortex and hippocampus, even after adjustment for neocortical Aβ load, supporting a direct contribution of APOE ε4 to early tau vulnerability in regions critical for memory decline 164. This view is further strengthened by large-scale multicohort analyses showing that tau PET positivity varies systematically with age, Aβ status, APOE genotype, and sex, with APOE ε4 shifting the age of tau positivity to substantially earlier time points and amplifying tau burden especially in Aβ-positive individuals 165. In this clinical context, "Aβ-positive" and "Aβ-negative" status refers to whether an individual's amyloid burden exceeds a pre-established biomarker threshold (e.g., cohort-specific cut-offs for PET SUVR or CSF Aβ42/Aβ40 ratios) rather than the absolute presence or absence of the protein 165. Large-scale studies show that a significant portion of cognitively unimpaired elderly individuals remain below these thresholds (Aβ-negative), representing a biological state distinct from the Alzheimer's pathological continuum 166.

In parallel, fluid biomarker studies suggest that APOE ε4 may accelerate tau-related pathophysiology long before clinical onset. Plasma p-tau181 becomes elevated many years earlier in ε4 carriers than in non-carriers, and this increase is accompanied by progressive hippocampal functional abnormalities, including early hyperconnectivity followed by later hypofunction and memory decline 167. Together, these findings argue that APOE genotype is not simply a modifier of amyloid deposition, but an upstream determinant of the timing, regional distribution, and clinical consequences of tau pathology.

Importantly, the effects of APOE on tau are not uniformly detrimental across all isoforms or disease contexts. Although APOE ε2 is generally considered protective in AD, evidence from primary tauopathies suggests a more complex biology. In PSP and CBD, APOE ε2 is associated with increased tau pathology and higher disease risk, indicating that APOE isoforms exert context-dependent effects that differ between Aβ-driven and primary tau-driven disorders 168. This distinction is highly relevant for review structure, because it suggests that the impact of APOE on tau cannot be understood solely through AD amyloid frameworks but must also be interpreted in terms of isoform-specific interactions with tau itself and with the lipid environment in which tau pathology develops.

### 6.2. Kinase Regulation and Isoform-Specific Tau Hyperphosphorylation

At the mechanistic level, APOE isoforms appear to influence tau pathology through at least two interconnected routes: direct or indirect regulation of tau handling, and modulation of kinase pathways that control tau phosphorylation. Early biochemical studies showed that APOE3 can bind tau and related microtubule-associated proteins, whereas APOE4 lacks this interaction, suggesting that isoform-specific structural properties may alter microtubule stability or tau self-assembly 169. More recent work has complicated the picture by showing that APOE2 can also exacerbate tau pathology under certain conditions, particularly in primary tauopathy, where non-lipidated APOE2 appears capable of forming disulfide-linked complexes with tau more efficiently than APOE3, while APOE4 shows little direct binding 168. These observations suggest that direct APOE-tau interactions are not simply “protective versus toxic” by isoform, but are strongly conditioned by biochemical state, especially lipidation.

Lipidation likely acts as a functional switch that determines whether APOE primarily participates in receptor-mediated signaling and lipid transport or instead becomes available for aberrant protein-protein interactions that favor tau pathology. When adequately lipidated, APOE engages receptors such as APOER2/VLDLR in signaling pathways linked to synaptic maintenance and repression of tau-phosphorylating kinases. In this context, APOE-dependent control of Reelin-ApoER2 signaling is especially relevant because it converges on GSK3β, one of the principal kinases involved in pathological tau phosphorylation. As reviewed in the tau kinase literature, dysregulated GSK3β activity is a major driver of tau hyperphosphorylation and neurodegeneration 170. A related connection arises from APP processing: the APP intracellular domain (AICD), generated by β- and γ-secretase cleavage, can activate GSK3β and thereby promote phosphorylation of tau and CRMP2 25. Thus, when APOE lipid homeostasis is impaired, the resulting shift in APP processing and receptor signaling may converge on GSK3β-dependent tau dysregulation.

Human cellular models further support a toxic gain-of-function model for APOE4 in tau pathology. Gene-editing studies in hiPSC-derived AD neurons reveal that converting APOE4 to APOE3 (via ZFN-mediated editing of the Arg112 locus to Cys112) significantly reduces p-tau levels, whereas the presence of APOE4 elevates p-tau compared to isogenic APOE3 or APOE-null controls 171. Furthermore, small-molecule correction of the APOE4 structure also ameliorates this phenotype, indicating that APOE4 drives tau pathology through intrinsic structural properties rather than merely through loss of APOE3 function 171.

Consistent with this interpretation, in vivo studies have shown that APOE4 markedly worsens tau-mediated neurodegeneration even in the absence of Aβ, whereas APOE knockout is strongly protective 172. Altogether, these findings support a model in which lipidated APOE participates in homeostatic receptor signaling and lipid trafficking, whereas insufficiently lipidated or structurally pathogenic APOE isoforms, particularly APOE4 and in some settings APOE2, shift the system toward kinase activation, aberrant tau handling, and neuronal injury.

### 6.3. Glial Responses, Receptor Competition, and the Propagation of Tau

Beyond neuronal tau phosphorylation, APOE lipidation also appears to shape the extracellular and glial environment in which tau accumulates, propagates, and causes neurodegeneration. One important mechanism involves cell-surface heparan sulfate proteoglycans and LRP1-mediated uptake pathways. ApoE recognizes AD-associated 3-O-sulfated heparan sulfate, and the binding affinity follows the order APOE4 > APOE3 > APOE2/Christchurch, paralleling AD risk 173. Since both Aβ and tau utilize cell-surface HSPGs (e.g., Syndecan-3) as essential co-receptors for internalization via micropinocytosis 174, APOE isoforms may compete for these binding sites, thereby modulating the cell-to-cell propagation of proteopathic seeds. Mechanistically, HSPG-mediated uptake of oligomeric Aβ42 involves its clustering in cholesterol-rich lipid rafts followed by the activation of the small GTPase Rac1, which drives macropinocytic internalization 175. Thus, the high-affinity interaction of APOE4 with HSPGs may not only disrupt Aβ clearance but also facilitate its pathological cellular entry. LRP1 remains a major regulator of tau uptake and spread, and alterations in APOE-HSPG-LRP1 competition could therefore influence whether extracellular tau is internalized for clearance or remains available for pathological seeding and propagation 176. These data fit well with the broader concept that tau spreads along neuronal communication networks, while glial and receptor-mediated processes modulate the efficiency and tissue specificity of that spread 177.

However, tau propagation is not purely a neuron-autonomous phenomenon. Increasing evidence indicates that microglia are major effectors of APOE-dependent tau neurotoxicity. In tauopathy mouse models, depletion of microglia almost completely prevents APOE-dependent neurodegeneration, demonstrating that pathological tau requires a microglial inflammatory program to translate into overt tissue loss 178. This idea is extended by work showing that gut microbiota and short-chain fatty acids modulate tau pathology in an APOE isoform- and sex-dependent manner, again emphasizing that APOE shapes the inflammatory milieu in which tau pathology progresses 179.

Within this framework, APOE lipidation emerges as a particularly important determinant of glial state. In P301S/APOE4 models, APOE4 promotes glial lipid droplet accumulation, disrupts cholesterol metabolism, and worsens neurodegeneration, whereas enhancing ABCA1-dependent lipid efflux, either genetically or through LXR agonism, reduces glial lipid accumulation, attenuates tau pathology, and protects against neurodegeneration 180. These findings strongly support the idea that insufficient APOE lipidation is not a secondary epiphenomenon, but a mechanistic contributor to tau toxicity through impaired glial lipid handling. In the same conceptual direction, lowering brain ApoE4 levels with antisense oligonucleotides reduces p-tau accumulation, suppresses inflammatory signaling, and limits neurodegeneration in tauopathy mice 181. Likewise, overexpression of LDLR lowers brain ApoE levels and ameliorates tau-associated neurodegeneration, further indicating that the amount, receptor handling, and lipidation-related trafficking state of ApoE are all relevant to tau pathogenesis 23.

Taken together, these studies support a unifying model in which APOE isoforms regulate tau pathology at multiple levels, but APOE lipidation is the key integrative node. By controlling receptor engagement, kinase signaling, glial lipid metabolism, inflammatory activation, and extracellular tau handling, APOE lipidation may determine whether APOE functions in a homeostatic, protective manner or instead amplifies tau phosphorylation, spread, and neurodegeneration.

## 7. Therapeutic Strategies Targeting APOE Lipidation

### 7.1. Direct Enhancement of APOE Lipidation through the LXR/RXR-ABCA1 axis

Among currently available approaches, the most direct strategy to enhance APOE lipidation is to activate the LXR/RXR-ABCA1 pathway, thereby increasing cholesterol efflux and loading APOE with lipids. This rationale is mechanistically attractive because APOE lipidation is not merely a biochemical modification of the carrier protein; rather, it determines whether APOE can efficiently support cholesterol redistribution, maintain membrane homeostasis, and facilitate Aβ clearance. The broader biological plausibility of this approach is reinforced by the demonstration that pharmacologic LXR activation promotes reverse cholesterol transport in vivo, with induction of ABCA1, ABCG1, and related sterol-handling genes 182. In the CNS context, this same transcriptional program is highly relevant because ABCA1-dependent lipidation is a key upstream determinant of APOE function in glia and extracellular Aβ homeostasis.

This mechanistic framework is supported by preclinical AD studies showing that increased levels of lipidated APOE enhance the proteolytic degradation of soluble Aβ. In particular, ApoE-facilitated Aβ degradation by neprilysin-related pathways in microglia and by IDE extracellularly depends strongly on APOE lipidation status, and LXR activation with GW3965 reduced cerebral Aβ burden while improving contextual memory in Tg2576 mice 19. These findings are important because they position APOE lipidation upstream of multiple Aβ-removal routes rather than restricting its role to simple carrier-mediated transport. Thus, from a therapeutic perspective, LXR agonists may work not only by increasing APOE abundance, but more importantly by shifting APOE toward a functionally lipidated state that is more competent for Aβ detoxification and brain cholesterol redistribution.

However, first-generation nuclear receptor agonists have proven difficult to translate. RXR agonism with bexarotene (a selective RXR agonist or "rexinoid") provided an important clinical proof-of-concept test of this pathway. Because RXR serves as a promiscuous heterodimerization partner for multiple nuclear receptors, including not only LXR but also the peroxisome proliferator-activated receptor (PPAR) family, rexinoids can theoretically activate both LXR/RXR and PPAR/RXR transcriptional programs, both of which contribute to the regulation of ABCA1 and APOE expression. Despite this broad mechanistic potential, the overall human trial outcome was negative. In a randomized placebo-controlled study of moderate AD, bexarotene did not significantly reduce amyloid burden in the overall cohort, although a genotype-stratified signal was observed in APOE4 non-carriers 182; by contrast, APOE4 carriers showed no measurable amyloid response 183. The importance of the PPAR/RXR axis is further highlighted by studies of selective PPAR agonists and natural modulators. PPARγ activation (e.g., via pioglitazone 184 or the soy isoflavone genistein 185) promotes the APOE-dependent clearance of Aβ by transcriptionally upregulating both APOE and ABCA1 in astrocytes and microglia. Mechanistically, this involves a PPARγ-LXRα-ABCA1 regulatory cascade 186 that enhances cholesterol efflux and APOE lipidation. Similarly, activation of PPARδ (via agonists like GW0742) can reduce neuroinflammation, although its impact on functional recovery remains complex; for instance, GW0742 effectively inhibits microglial activation but may fail to restore neurogenesis or prevent cognitive deficits in certain brain injury contexts 187. Indeed, the rapid Aβ clearance and cognitive recovery initially observed with the RXR agonist bexarotene in preclinical models 188 are thought to depend on the coordinated activation of both LXR/RXR and PPAR/RXR pathways. Despite these compelling preclinical data, large-scale clinical trials of PPARγ agonists (e.g., the TOMMORROW trial) failed to show cognitive benefit, likely due to similar challenges of metabolic side effects, blood-brain barrier penetration, and late intervention timing. These results suggest that while the LXR/RXR and PPAR/RXR pathways are biologically actionable molecular nodes, clinical benefit is unlikely to emerge from non-selective activation alone, particularly when metabolic toxicity, inadequate CNS selectivity, and genotype-dependent response heterogeneity are not addressed.

Recent studies therefore point toward a more refined second generation of lipidation-enhancing compounds. For instance, the LXR agonist GW3965 was recently shown to ameliorate both Tau pathology and ApoE4-linked glial lipid accumulation 180. A notable example is the brain-penetrant LXRβ-selective agonist CE9A215 189, which preferentially activates LXRβ without appreciable LXRα activation, crosses the blood-brain barrier, and in 3xTg-AD mice reduces Aβ deposition, phosphorylated tau, and neuroinflammation while improving behavioral outcomes. Similarly, the semi-synthetic agonist 22-ketositosterol 190 appears to induce cholesterol-efflux programs with less lipogenic liability and shows neuroprotective effects in APPswe/PS1ΔE9 mice, particularly by reducing glial inflammatory markers and preserving spatial memory, even though its effect on plaque burden is limited. Moreover, peptide mimetics such as CS-6253 that target the ABCA1 transporter represent an alternative strategy to bypass the systemic liabilities of nuclear receptor activation 141. Together, these newer studies suggest that the future of APOE lipidation therapy will likely depend less on maximal pathway activation and more on achieving a therapeutically useful balance among CNS exposure, LXRβ selectivity, and metabolic safety.

### 7.2. Correcting APOE4-Specific Lipidation Defects rather than Globally Increasing APOE

A second, and in many ways more precision-oriented, therapeutic direction is to correct the APOE4-specific cellular defects that impair lipidation. This is important because APOE4 pathology is not simply a quantitative deficiency of APOE, but a qualitative failure to generate and maintain properly lipidated APOE particles. In astrocytes, ApoE4 promotes ABCA1 aggregation and decreases ABCA1 recycling to the plasma membrane, thereby reducing cholesterol efflux and generating lipid-poor, aggregation-prone APOE particles 141. This shifts the therapeutic focus from generic transcriptional upregulation of ABCA1 to restoration of ABCA1 membrane trafficking and recycling. Conceptually, such an approach may be especially valuable in APOE4 carriers, where the relevant defect lies not only in the amount of transporter expression but also in transporter localization and function.

This APOE4-specific framework also helps connect lipidation-targeted therapy to APP processing. ApoE4, particularly in a poorly lipidated or structurally pathogenic state, enhances Aβ production by altering APP trafficking through an LRP-dependent mechanism, and disruption of ApoE4 domain interaction can abolish this excess Aβ production 136. Accordingly, structure correctors that convert ApoE4 toward an ApoE3-like conformation may complement lipidation-based therapies by reducing the generation of lipid-poor, dysfunctional ApoE4 particles while also attenuating their pro-amyloidogenic influence on APP processing. In this view, “targeting APOE lipidation” should be interpreted broadly: not only increasing lipid loading, but also repairing the structural and trafficking context required for lipidation to translate into functional benefit.

The relevance of this broader interpretation is reinforced by recent work in tauopathy models. In P301S/ApoE4 mice, glial cholesterol ester accumulation, lysosomal dysfunction, and neuroinflammation are markedly worsened by ApoE4, whereas increasing lipid efflux through an LXR agonist or Abca1 overexpression attenuates tau pathology, reactive gliosis, synaptic loss, and neurodegeneration 180. These data extend the therapeutic scope of APOE lipidation beyond Aβ clearance alone. They suggest that restoring lipid efflux and APOE lipidation may reprogram a pathogenic glial state that feeds both amyloid and tau pathology. For a review centered on lipidation, this point is important: the value of these interventions may lie as much in resetting glial lipid handling and synaptic support as in directly lowering plaque burden.

### 7.3. Early Intervention, Genotype Stratification, and Adjunctive Metabolic Support

Despite strong mechanistic rationale, successful translation will require much stricter alignment between therapeutic design and disease biology. CNS drug development has repeatedly failed because compounds with plausible targets often suffer from poor brain delivery, limited predictive validity of animal models, and late-stage intervention in biologically advanced disease 191. For APOE lipidation-directed therapies, these concerns are especially relevant, as their efficacy is highly dependent on the disease stage. A temporal framing clarifies why therapeutic responses may vary significantly 69: During the preclinical stage, correcting APOE lipidation can maintain baseline membrane homeostasis and optimize Aβ clearance, offering the greatest preventive potential 158. As the disease progresses to the early amyloid stage (biomarker-positive but minimally symptomatic), enhancing APOE lipidation could still effectively redirect soluble Aβ toward degradation and prevent downstream neurotoxicity, representing the optimal therapeutic window for single-agent lipidation therapies. However, once patients reach the symptomatic stage with established amyloid and tau pathologies, the primary goal shifts from prevention to merely mitigating ongoing glial lipid dysregulation; here, efficacy may be blunted, likely requiring combination approaches 69. Finally, in the late stage of severe dementia characterized by irreversible structural brain loss, merely restoring lipid transport is unlikely to yield meaningful clinical benefit. This timing argument aligns with the NIA-AA biological framework, which emphasizes biomarker-defined disease stages 192. In practice, this means that lipidation-targeted interventions will be most effective in the early amyloid phase, particularly for APOE4 carriers, rather than in established, late-stage dementia.

Clinical development should likewise incorporate genotype-aware trial design from the outset. ApoE genotype influences disease mechanisms, progression rates, and, in some settings, treatment response and safety, arguing that it should be included as a stratification factor or covariate in AD trials 166. The bexarotene experience further illustrates why this is not a statistical detail but a biological necessity: the amyloid signal emerged in APOE4 non-carriers, whereas APOE4 carriers did not respond detectably. Future trials of APOE lipidation therapies should therefore be explicitly designed around genotype, target engagement biomarkers, and stage-specific enrollment rather than relying on pooled analyses across biologically heterogeneous populations.

Finally, indirect metabolic interventions may best be viewed as adjuncts rather than primary lipidation therapies. Observational and re-analysis studies suggest that statins may confer cognitive or risk-related benefit, potentially with greater effects in APOE4-defined subgroups, especially for brain-penetrant lipophilic agents 193. Mechanistically, this idea is plausible because lowering cholesterol can favor non-amyloidogenic APP processing through ADAM10 80. Likewise, Mediterranean-style dietary interventions improve systemic lipid and inflammatory profiles, providing a permissive metabolic context for healthier lipid handling 194. However, neither statins nor diet should be presented as direct proof of APOE lipidation rescue in the brain. Rather, they are best framed as supportive strategies that may modulate the upstream lipid environment within which APOE lipidation-targeted drugs operate. Looking ahead, one of the main bottlenecks remains CNS delivery, and emerging brain-delivery platforms such as engineered exosomes may eventually help transport small molecules, nucleic acids, or APOE4-corrective therapeutics across the BBB more efficiently 195.

Overall, therapeutic strategies targeting APOE lipidation are most compelling when viewed not as a single drug class, but as a mechanistically connected intervention space. Direct LXRβ/ABCA1 activation aims to increase the supply of functional lipidated APOE; APOE4-specific trafficking and structure-correcting approaches seek to restore the cellular machinery that generates such particles; and metabolic or delivery-oriented adjuncts may improve the context in which these interventions work. The field is now moving from proof-of-principle toward precision design. The major next steps are to develop brain-penetrant and metabolically safe modulators, establish biomarkers that report APOE lipidation or ABCA1 engagement in vivo, and test these agents in genotype-stratified, biomarker-defined early AD populations.

## 8. Conclusion

This review highlights the importance of APOE lipidation as a key molecular factor in the development of AD, acting as the crucial link between genetic risk and downstream neurodegenerative processes. Rather than acting like a fixed genetic risk modifier such as APOE4, APOE lipidation dynamically influences protein conformation, intracellular transport, receptor binding, and lipid-protein interactions, thereby coordinating multiple pathways involved in the disease. In this framework, adequately lipidated APOE, particularly APOE2 and APOE3, supports cholesterol redistribution, preserves membrane organization, facilitates Aβ clearance, and helps maintain synaptic integrity. In contrast, poorly lipidated APOE4 is more prone to aggregation, less efficient in lipid transport and receptor-mediated signaling, and more likely to shift the brain environment toward amyloidogenic APP processing, impaired Aβ clearance, glial dysfunction, neuroinflammation, and tau-related neurodegeneration.

A major theme emerging from this review is that APOE lipidation links lipid metabolism to the two core pathological axes of AD, namely Aβ and tau. In the context of Aβ pathology, APOE isoforms and lipidation state regulate multiple interconnected processes spanning Aβ production, aggregation, and clearance. At the level of APP processing, APOE lipidation modulates neuronal membrane composition and lipid raft organization, thereby influencing the partitioning of APP and secretases and shifting the balance between non-amyloidogenic and amyloidogenic pathways, while lipidation-dependent receptor signaling and trafficking further regulate APP expression and endocytic processing. Beyond production, APOE also shapes extracellular aggregation, vascular and cellular clearance, and proteolytic degradation of Aβ. Among these processes, the evidence is strongest for a critical role of APOE lipidation in Aβ clearance, where ABCA1-dependent lipid loading determines whether APOE can effectively support trafficking and degradation pathways, whereas its direct role in Aβ aggregation remains less clearly defined and warrants further investigation. In tau pathology, APOE lipidation functions as an integrative regulator of receptor signaling, kinase activation, glial lipid homeostasis, inflammatory state, and extracellular tau handling, thereby influencing not only tau phosphorylation but also tau spread and neurotoxicity.

These mechanistic insights also carry important translational implications. Therapeutic strategies that enhance APOE lipidation or correct APOE4-specific lipidation defects may offer a way to intervene upstream of multiple downstream pathologies simultaneously, rather than targeting Aβ or tau in isolation. Preclinical studies targeting the LXR/RXR-ABCA1 axis, restoring ABCA1 function, or correcting APOE4 structure support the idea that improving APOE lipidation can reduce amyloid burden, attenuate tau pathology, normalize glial responses, and protect synaptic function. At the same time, the mixed clinical experience with earlier pathway activators underscores that successful translation will require brain-penetrant, metabolically safe, and genotype-aware approaches, together with biomarkers capable of reporting APOE lipidation status in vivo.

Overall, the available evidence supports a model in which APOE lipidation is a key molecular node connecting APOE genotype to membrane biology, synaptic vulnerability, Aβ dyshomeostasis, tau pathology, and neuroinflammation in AD. Further clarification of how lipidation differentially shapes APOE structure and function across disease stages, cell types, and isoforms will be essential. A deeper mechanistic and translational understanding of this process may not only refine our view of APOE biology but also open the door to precision therapies aimed at restoring lipid homeostasis and synaptic resilience in Alzheimer's disease.

## Abbreviations

AD: Alzheimer's disease
Aβ: amyloid-β
APOE: apolipoprotein E
ABCA1: ATP-binding cassette transporter A1
ABCG1: ATP-binding cassette transporter G1
ABCA7: ATP-binding cassette transporter A7
ABCB1: ATP-binding cassette transporter B1
ABC: ATP-binding cassette
AICD: APP intracellular domain
ADAM10: a disintegrin and metalloproteinase domain-containing protein 10
AMPAR: α-amino-3-hydroxy-5-methyl-4-isoxazolepropionic acid receptor
AEP: asparagine endopeptidase
AP-1: activator protein 1
APP: amyloid precursor protein
APLP2: amyloid precursor-like protein 2
APOER2: apolipoprotein E receptor 2
APPsα: soluble amyloid precursor protein alpha
ARF6: ADP-ribosylation factor 6
BACE1: β-site amyloid precursor protein cleaving enzyme 1
BBB: blood-brain barrier
CBD: corticobasal degeneration
Cer: ceramide
cGAS-STING-IFN: cyclic GMP-AMP synthase-stimulator of interferon genes-interferon
CLU: clusterin
CNS: central nervous system
CRMP2: collapsin response mediator protein 2
CSF: cerebrospinal fluid
Dab1: disabled-1
DAP12: DNAX-activating protein of 12 kDa
DLK: dual leucine zipper kinase
ER: endoplasmic reticulum
ERK1/2: extracellular signal-regulated kinase 1/2
GCase: glucocerebrosidase
GM1: monosialotetrahexosylganglioside
GSK3β: glycogen synthase kinase 3β
GTs: glycosyltransferases
HDL: high-density lipoprotein
hiPSC: human induced pluripotent stem cell
HSPGs: heparan sulfate proteoglycans
IDE: insulin-degrading enzyme
ISF: interstitial fluid
LC: locus coeruleus
LDL: low-density lipoprotein
LDLR: low-density lipoprotein receptor
LRP: low-density lipoprotein receptor-related protein
LRP1: low-density lipoprotein receptor-related protein 1
LTP: long-term potentiation
LXR: liver X receptor
LXRα: liver X receptor alpha
LXRβ: liver X receptor beta
MAPK: mitogen-activated protein kinase
MCI: mild cognitive impairment
MKK7: mitogen-activated protein kinase kinase 7
NEP: neprilysin
Neu-ases: neuraminidases
NIA-AA: National Institute on Aging-Alzheimer's Association
NMDAR: N-methyl-D-aspartate receptor
NVU: neurovascular unit
PET: positron emission tomography
P-gp: P-glycoprotein
PI3K: phosphatidylinositol-3-kinase
PICALM: phosphatidylinositol-binding clathrin assembly protein
PKB/Akt: protein kinase B
PPAR: peroxisome proliferator-activated receptor
PPARγ: peroxisome proliferator-activated receptor gamma
PPARδ: peroxisome proliferator-activated receptor delta
PSEN1: presenilin 1
PSP: progressive supranuclear palsy
pTau: phosphorylated tau
Rac1: Ras-related C3 botulinum toxin substrate 1
RXR: retinoid X receptor
SFK: Src family tyrosine kinases
SORL1: sortilin-related receptor 1
SUVR: standardized uptake value ratio
TREM2: triggering receptor expressed on myeloid cells 2
VLDL: very low-density lipoprotein
VLDLR: very low-density lipoprotein receptor
ZFN: zinc finger nuclease
βGal-ase: β-galactosidase
βHex-ase: β-hexosaminidase
